# Irrationality in humans and creativity in AI

**DOI:** 10.3389/frai.2025.1579704

**Published:** 2025-06-20

**Authors:** Olha Sobetska

**Affiliations:** Centre Leo Apostel (CLEA), Vrije Universiteit Brussel, Brussels, Belgium

**Keywords:** irrationality, decision-making, creativity, artificial intelligence, conjunction fallacy, methodological fallacy, cognition

## Abstract

This manuscript explores how human irrationality in decision-making can contribute to artificial intelligence (AI) development, particularly in the domain of creativity. While irrational behavior is typically seen as a cognitive flaw, we argue that certain forms of irrationality, such as those demonstrated by the conjunction fallacy (CF), may represent context-sensitive reasoning that reveals creative problem-solving. Traditional AI research has primarily focused on rational, logic-driven models, overlooking the productive role of non-linear and seemingly illogical human thinking in generating novel insights. Drawing on interdisciplinary insights and recent neuroscientific findings, particularly the interaction of the Default Mode, Executive Control, and Salience Networks, we propose a model that integrates both rational and irrational cognitive dynamics. This framework may inform the design of AI systems that are more adaptive, context-aware, and capable of emulating human-like creativity.

## Introduction

The rise of artificial intelligence (AI) has ushered in a new era of both technological advances and a deeper understanding of mind and reasoning (Lake et al., 2017; Poldrack and Yarkoni, 2016; Mnih et al., 2015; Zhang et al., 2025). While Deep Neural Networks (DNNs) mimic neural structures in the brain, researchers use this statistical algorithm to study actual neuronal mechanisms (Marblestone et al., 2016; Kriegeskorte and Douglas, 2018). AI thus opens twin horizons—technological and cognitive—that complement each other. Given that cognitive findings can significantly advance AI development, it makes sense to update established cognitive science paradigms with recent discoveries in neuroscience and potentially use them as a source for AI improvement (Hassabis et al., 2017).

This manuscript offers a novel contribution by re-evaluating the role of irrationality in cognitive science. It proposes that certain forms of irrational decision-making—long treated as fallacies or biases—may actually support creative thinking. Building on recent findings in neuroscience, we aim to update definitions of rational and irrational behavior and (re)connect ideas from cognitive psychology (e.g., heuristics and biases), scientific methodology (e.g., fallacies in reasoning), and brain network dynamics. These elements are brought together as an attempt to form a unified framework for modeling human-like creativity in AI. Unlike traditional approaches that view irrationality as a flaw in reasoning and AI models that prioritize logic, optimization, and consistency, we suggest that irrationality, under some conditions, can be a valuable source of creative insight.

Why could irrationality be important for AI, especially generative AI? Recent studies have shown that AI has or potentially may have a “creativity crisis.” Hataya et al. (2023) demonstrated that a classical image generation task (specifically generating an elephant) became less creative as algorithms increasingly repeated patterns from reference images. This decline can stem from a loop where previously generated images (outputs) become reference materials (inputs) for newer algorithms. This phenomenon can be described as the “ouroboros problem,” an analogy to the ancient symbol of a self-consuming snake. The ouroboros problem extends beyond image generation to language models as well. Guo et al. (2023) established this as a widespread issue in language models, noting that output quality and diversity decrease when language generation tasks require a higher degree of creativity.

Moreover, Laverghetta et al. (2025) conducted a comparative analysis between human experts and Large Language Models (LLMs) in evaluating outputs by their creativity. Their findings demonstrated that LLMs can accurately predict human creativity assessments, however, the underlying mechanisms and evaluation criteria used by these models remain unspecified:

"LLMs can achieve impressive accuracy in predicting human creativity assessments, yet we know little about how they arrive at their judgments, what features they prioritize, or whether their evaluation strategies align with those of human experts." (Laverghetta et al., 2025)

Thus, there is a lack of creativity or understanding of how machines interpret creativity at least for some kinds of tasks. Considering this problem, we aim to define creativity with its complexity and discuss how such a definition can contribute to AI development. First, we examine one of the main concepts in decision-making theory: the conjunction fallacy, illustrating the complexity of human decision-making and its relation to strict probabilistic norms. More precisely, CF challenges traditional probability theory by showing how human decision-making often deviates from purely rational calculations (based on Kolmogorov’s probability theory). Research has extensively demonstrated that individuals frequently assign higher probabilities to a conjunction of specific conditions than to a single event [P(a) & P(b) > P(a)]—violating basic laws of probability. Tversky and Kahneman (1983) labeled such behavior as a fallacy (irrational). But is it truly a fallacy? Above mathematical contra-argument, which will be discussed in the next chapter, one intuitive argument suggests that people who do not work directly with statistics or probability theory should not be expected to apply its calculations to real-life examples. Furthermore, even participants with sophisticated statistical knowledge in Kahneman and Tversky’s experiments committed the conjunction “fallacy.” To understand the roots of this seemingly irrational behavior, we will examine key critical points of the CF and contexts in which such behavior, while not based on classical probability theory, can be justified by reasoning in a particular context.

While rational thinking is necessary and sometimes even vital in many areas, such as legal, financial, and medical decision-making etc., irrationality may not always be disadvantageous. What is classified as “irrational” behavior can contribute significantly to creative problem-solving, especially in situations where conventional rules and protocols are unable to lead to novel solutions. This is evident in scientific research, where strict adherence to rational and/or statistical methods without considering contextual nuances can paradoxically lead to less meaningful or even unreliable results. Typically, such a problem is labeled as a methodological fallacy in the research context, which will be discussed in detail in the next section, taking concrete examples from sociology, biomedicine, and linguistics. While the methods and formulas may be performed technically correctly, the results and their interpretation may be biased or may not provide sufficient insights into the subject matter under study, mostly due to its complexity. So, if AI struggles with creative reasoning, could an examination of human irrationality give some insights to improve it?

Trying to answer this, we will examine the concept of creativity, both from psychological and neurobiological perspectives. Studies in neuroscience suggest that creativity arises from a dynamic interplay between the Default Mode Network (DMN), responsible for free-flowing thought, and the Executive Control Network (ECN) or focused mode, which helps refine and structure those ideas. Considering this insight and some cognitive theories, we will define creativity as a balance between the rational and the irrational and discuss how it could improve AI’s ability to generate novel and meaningful outputs.

## Conjunction fallacy

The Conjunction Fallacy is a phenomenon in cognitive psychology defined by Kahneman, Nobel Prize winner in economics, two scientists who contributed enormously to the development of the field of behavioral economics and decision-making theories (Morier and Borgida, 1984; Tversky and Kahneman, 1983; Moro, 2009; Gigerenzer, 1996; Tentori et al., 2004). This phenomenon belongs to a family of cognitive biases that includes Prospect Theory, the Allais Paradox, and the Framing Effect—all examining how context, risk, and uncertainty shape the assessment of probabilities in decision-making (Tversky and Kahneman, 1974). While each of these phenomena deserves a separate study, this manuscript focuses solely on the conjunction fallacy, which provides a sufficient foundation for a thorough discussion.

### Conjunction fallacy: concept

The conjunction fallacy can be explained by its name. Conjunction means judgment of the probability of two events occurring together [P(A) & P(B)] higher than the probability of one of the constituent events occurring alone [P(A) or P(B)]. Fallacy means, according to Kahneman and Tversky, that such a judgment is a violation of probability theory, which states that the probability of two events occurring together cannot be greater than the probability of either event occurring individually. The way to test the CF, respondents were asked to solve a task, which was named the “Linda problem.” In this example, participants are given a description of Linda: “Linda is 31 years old, single, outspoken, and very bright. She majored in philosophy. As a student, she was deeply concerned with issues of discrimination and social justice, and also participated in anti-nuclear demonstrations.”

Next, they are asked to rank the probability of various statements about Linda, including: (A) Linda is a bank teller; (B) Linda is a bank teller and is active in the feminist movement.

In this design, which was named a transparent test (p. 299), 85% of respondents ranked B (the conjunction) as more probable than A (the single event). When researchers manipulated the response format by changing the probability assessment to a scale from 1 to 9, the conjunction fallacy still persisted among the majority (82%), with participants rating A at 3.5 and A&B at 5.6. In addition to Linda’s task, there were other tasks with a male character named Bill to test a possible gender bias, there were experiments with a wider range of response options, and experiments with different levels of statistical knowledge among respondents (from sophisticated to minimal) - all of these manipulations did not solve the CF problem.

### Conjunction fallacy: critical points

“Outside the laboratory, however, outcomes and probabilities are rarely known with certainty and served up to the decision-maker on a platter.” (Hertwig and Gigerenzer, 2011, p. 1)

The research of Kahneman and Tversky demonstrated very clearly that humans often make decisions based on cognitive biases and heuristics rather than a probabilistic approach. The researchers manipulated words, and their positions in a sentence, study design, answer type, male/female figures in the vignettes, and more features to demonstrate that humans tend to deviate from probability calculation. However, such a deviation could be called irrational if one takes probability theory as a reference, which is not the case in real-life events. Moreover, what is the number of people who keep assumptions of classical probability theory every day and apply it by making real-life decisions?

In general, criticism of the CF could be divided into two elements: method and study design.

On method: Kolmogorov probability theory (Kolmogorov, 1956) implies the assumption of infinity, meaning that a situation must be repeatable an infinite number of times. However, the Linda example cannot satisfy this assumption. In fact, most real-life scenarios lack this ability per se. Therefore, applying a method with such a condition is inadequate for analyzing situations that cannot meet this requirement. Alternative methods that eliminate infinity requirements are the Bayesian approach and other mathematical methods, such as fuzzy logic and possibility theory (Jaynes, 2003; Zadeh, 1965; Dubois and Prade, 1988).

On study design: The Linda task lacks an indication that it should be solved by operating with a purely probabilistic approach. Among other CF issues, this critical point was clearly demonstrated in Hertwig and Gigerenzer (1999) study—a highly relevant critique of the conjunction fallacy existing in the literature. The researchers found that the conjunction fallacy decreases significantly when tasks are formulated using probabilistic language, which serves as a signal of the mathematical context to respondents. More precisely, they first tested and proved that the initial context of the task was not taken in mathematical terms among the respondents. Next, they studied precisely which words would be associated with a mathematical context for the Linda problem. Finally, they applied these semantic findings to test the CF with a clear indication of the probabilistic context, using a language of frequency. After doing this, the CF was committed to only 13% of respondents (the original result was 82%). This has demonstrated that one can set up a context or method to solve the issue, indicating this directly and not post-factum. This phenomenon is consistent with the concept of framing in psychology (Tversky and Kahneman, 1981) because the way Linda’s problem is presented influences respondents’ interpretations and decision-making strategies, demonstrating that changes in wording can affect the outcome.

The next study by Polakow et al. (2021) has shown that the CF rate can also drop if one designs the task by choosing between two rank orders of options instead of freely ranked multiple statements (free ranking), with 61 and 32%, respectively. Participants made fewer conjunction errors when providing probability estimations compared to making categorical choices. This suggests that requiring individuals to think in terms of numerical probabilities can reduce the likelihood of the conjunction fallacy.

The study design could be reviewed not only internally (within the original paper), but also externally (further CF experiments). Since the seminal papers were published in 1983, there have been thousands of new studies and experiments conducted, even in recent years. This may signal two issues:

1. The misalignment between Linda’s scenario and the application of Kolmogorov probability theory is not obvious to the scientists conducting such experiments.
2. Such experiments are conducted without taking into account the critical points on the study design described above (Veloz and Sobetska, 2023).

### Conjunction fallacy: what did we learn?

The analysis of the conjunction fallacy reveals more than just a cognitive bias. It exposes a fundamental issue in how problems are framed and how methods are applied. It can further be seen as a tendency to judge complex phenomena using tools without regard for contextual fit. Humans do not usually rely on probability theory to make life-relevant decisions, but they are able to solve a problem using probability theory if they are semantically given that context. As a result, applying classical probability theory to a real-life scenario, without ensuring that participants interpret it mathematically, leads to misleading conclusions about human rationality. This is especially relevant in creative problem-solving, where solutions may appear irrational by Kahneman’s and Tversky’s standards but are rational within their specific context. This insight thus motivates one to consider problem solving strictly within the nature of the problem, rather than manipulating it.

However, this type of mismatch is not limited to psychology but recurs across disciplines, where methodology can oversimplify the nature of the object under study. The next section explores this broader issue as the “methodological fallacy,” using examples from sociology, biomedicine, and linguistics to show how such misalignments affect reasoning and interpretation in diverse fields.

## Methodological fallacy

The misalignment between the nature of the object under study and the method that studies it can be found not only in the CF (Mugur-Schächter, 2002). This misalignment is a cross-disciplinary phenomenon, which is called a methodological fallacy.

### Sociology

“Perhaps it is not so wrong to compare a social scientist with a spy in a foreign country. A good scout not only reports obvious and desirable facts, but also hidden and unpleasant ones.” (Diekmann, 2018, p. 60)

In sociology, a methodological fallacy known as the ecological fallacy occurs when drawing conclusions about individuals based on group-level data. The term “ecological” refers to the collective dimension, and this concept was introduced by sociologist W. S. Robinson. In his extensive study (Robinson, 1950) examining literacy and foreign birth rates across U. S. states, Robinson demonstrated the difference between individual and collective correlations. He discovered a paradox: while states with higher proportions of foreign-born residents had higher literacy rates overall, individual-level data revealed the opposite trend. This contradiction points out the dangers of misinterpreting aggregate data and has made a huge contribution to the design of surveys and quantitative research methods.

Shortly: the ecological fallacy appears when one concludes (generalizes) from collective to individual levels.

Example: Supposedly, we have a hypothesis about alcohol consumption and life expectancy, so it can be formulated as follows:

*H1*: Countries with high alcohol consumption have a higher life expectancy.

**Figure 1:**
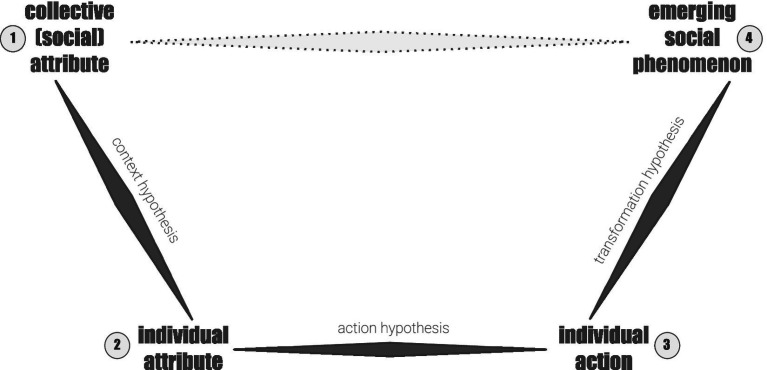
Model of ecological fallacy. Own representation, adapted initially from Coleman’s bathtub model (Coleman, 1990) and complemented by argumentations and models by Boudon (1979, 1980), Esser (1999), Lindenberg (1977) and Opp (2002). [1, 4]—collective (macro) levels, [2, 3]—individual (micro) levels.

Based on Figure 1, alcohol consumption is a collective (social) attribute [1] inside a country; an emerging social phenomenon here is a (collective) higher life expectancy [4]. Ecological fallacy is the conclusion that alcohol increases life expectancy [1 → 4] without testing alcohol consumption and life expectancy by individuals [2 → 3]. Theoretically, such countries could be wealthier, have better health care systems, healthier lifestyles in general, or, as an obvious example, invest better in sociological research, thereby improving the quality of population data.

To avoid ecological fallacy, we should build and test additional hypotheses:

[1 → 2] Context hypothesis: Which (collective) factors influence individual alcohol consumption behavior? For example, higher incomes and a higher standard of living in a country (Macro–Micro).

[2 → 3] Action/behavior hypothesis: Which individual factors lead to higher life expectancy? For example, a person with moderate consumption could be less affected in terms of health because other lifestyle factors (healthy diet, high-quality sleep, medical care, sport, good stress management, and social connections) offset the negative effects of alcohol (Micro–Micro).

[3 → 4] Transformation/aggregation hypothesis: How do individual decisions lead to emerging/aggregated social phenomena? A country with a high life expectancy may have many healthy people with moderate consumption and good medical care. In another country with lower life expectancy, health problems may be caused more by poverty, poor medical care, and work conditions than by low alcohol consumption (Micro–Macro).

### Biomedicine and statistical misinterpretation

Biomedical research is one area where misuse of the *p*-value is a frequent methodological fallacy (for details, see Steyerberg et al., 2018; Benjamin et al., 2018; Greenland et al., 2016; Gao, 2020; Gliner et al., 2001; Ioannidis, 2019; Benjamin and Berger, 2019). The p-value is a statistical measurement used to calculate the probability that an observed outcome occurs by chance, assuming that the null hypothesis (e.g., null treatment effect) is true. If a drug study has a *p* > 0.05 (a generally accepted level at which a result is considered nonsignificant), it may still have medical significance. In other words, the study may contribute to much of the research in other areas of medicine. Thus, the p-value should not be considered as a measure of success or failure, but rather a filtering tool (Sobetska, 2023). Moreover, this (un)significant outcome can be manipulated by sample size because the p-value function refers to it, and this is the second point where a methodological fallacy arises:

“(…) two studies into new treatments for a certain disease are published with only the p values, stating that the effect of the first drug was statistically not significant, while that of the second was. The naive reader might think that the second drug should be used, and the first one dismissed. However, the difference may have been the result of differences in sample size, and in reality the non-significant study may have had a more pronounced and clinically relevant effect, worthwhile to explore further, whereas the large study yielded a statistically significant result, but a clinically irrelevant effect.” (Rosendaal, 2016, p. 22)

Unlike *p*-values, confidence intervals provide a range of plausible values for the estimated effect, providing a sense of precision and uncertainty. As another alternative, effect sizes quantify the strength of a relationship or difference, allowing comparisons across studies and contexts. However, despite the availability of alternative methods and the restriction of the p-value test in scientific publications, the problem of understanding the null hypothesis significance test (NHST) remains at the educational and research levels (Haller and Kraus, 2002; Badenes-Ribera et al., 2015, 2016; Lyu et al., 2020; Lytsy et al., 2022; Sobetska, 2023).

### Linguistics

In linguistics, a highly relevant cognitive science field, the methodological fallacy occurs almost in the same way as in the example of sociology - in generalization. Evans and Levinson (2009) paper postulates the need to treat each language separately, which can be considered as a counterargument against the theory of universal grammar, promoted by Noam Chomsky. The researchers argue further that language emerges from a combination of cognitive processes, cultural evolution, and environmental adaptation, rather than having a universal grammar.

A key methodological fallacy arises when linguistic exceptions are treated as anomalies rather than as evidence against universality. For example, the Pirahã language lacks recursion (Everett, 2005), contradicting claims that it is a defining feature of human language (Hauser et al., 2002), yet many generative linguists have attempted to reinterpret the data rather than revise their models (Nevins et al., 2009). Similarly, languages such as Indonesian Riau (Gil, 2005) and Salish (Wiltschko, 2003) challenge assumed grammatical universals. Rather than imposing Indo-European structures on all languages, Evans and Levinson (2009) were motivated to apply a language diversity approach, recognizing linguistic variation (inconsistency) as fundamental. In other words, a bottom-up rather than top-down approach to linguistic theory, meaning that theories of language should be derived from extensive research and analysis of global linguistic diversity.

These few examples demonstrate the importance of matching the problem to the tool, and the ability to delve into the complexity of phenomena without simplifying results only to a computational and/or generalized level. Applying a straightforward, rational approach to inference can miss meaningful data points that can reveal the deeper nature of the objects being studied. However, due to the complexity of phenomena, this is irreversible in many cases. This irreversibility is due to both technical (methodological) and cognitive limitations. Therefore, recognizing and respecting both the computational and chaotic aspects of decision-making becomes essential for a deeper understanding of cognitive processes.

## Concept of creativity

“The subject of creativity has interdisciplinary appeal. This is true because the phenomenon to which the term creativity applies is the phenomenon of synthesizing knowledge. Hope for greater unification of knowledge lies in the continuance of studies of creativity.” (Rhodes, 1961, p. 310)

Defining creativity is in itself a complex and daring process. This section provides a brief overview of the evolution of creativity theory, but with full respect for its richness.

Historically, the foundation of creativity research is based on the framework, called the 4 P’s model, proposed by Rhodes (1961), where he set it through four dimensions:

1. *Person*—the relationship between creativity and individual traits, habits, intelligence, and personality. Rhodes argues further that a high intelligence level does not automatically mean the presence of creative skills, while this correlation can be seen in quick humor and complex temperament.
2. *Process*—mental operations and strategies used in creative thinking. As an example of such mental operations, he discusses stages of the thinking process of the German physicist and physiologist Hermann Helmholtz, which are preparation (observation and analysis), incubation (conscious and unconscious processing), illumination (solution emergence), and verification (testing).
3. *Press* (Environment)—perception and sensory of external influences such as culture and environmental needs, and a personal response to them. This process explains why great inventions sometimes arise from different minds that may live in societies with the same social needs and technical possibilities for their satisfaction.
4. *Product* (idea)—an outcome of creative efforts. Although this “P” is concluding, the author argues that research into the nature of the creative process can only go in one direction: from the product to the person and then to the process and the press:
  “Products are artifacts of thoughts. Through the study of artifacts, archeologists reconstruct the way of life of extinct peoples, officers of the law reconstruct the events leading up to a crime, and psychologists reconstruct the mental processes of inventing.” (Rhodes, 1961, p. 309)

Using this framework as a central model, the global research on creativity was split between these P’s (see Basadur et al., 2000; Runco and Albert, 2010; Parkhurst, 1999) for a comprehensive literature overview), so that some focused on attributes of creative personalities and cognitive traits, while others prioritized environmental factors. Thus, the main problem in defining creativity is whether to define creativity as an attribute or as a process. The standard definition of creativity proposed by Runco and Jaeger (2012) describes creativity as the ability to generate ideas, solutions, or products that are both original (novel) and efficient (utilitarian, relevant to the context, and aligned with values). Glăveanu and Beghetto (2021) extend the standard definition with some personal principles (soft skills) such as open-endedness, nonlinearity, pluri-perspectives, and future-orientation, criticizing that novelty and meaningfulness alone are not enough to define creativity. Besides, Kasof et al. (2007) add some significance to personal values in addition to motivation in the context of creativity.

In contrast, Green et al. (2024) make a distinction between creativity as an attribute and as a process. While the standard definition focuses on the evaluation of the creative product, their process-oriented approach defines creativity as “inner attention constrained by a generative purpose.” This means that creativity is not simply the creation of something new, but a dynamic interplay of attention, cognitive flexibility, and goal-directed idea generation.

So, how does this process emerge? This question can be explored from both theoretical and neurobiological perspectives. One of the relevant cognitive theories is the theory of divergent and convergent thinking, proposed by Guilford (1950, 1968). According to this theory, divergent thinking involves breaking rules and questioning traditional points of view, consequently generating multiple unique ideas or solutions to a given problem. Mednick (1962) also pointed out that creative products are formed through unconventional connections between seemingly unrelated concepts (like Einstein’s theory about space–time or juxtaposition). Runco and Jaeger (2012) contribute to this point with their argument that creativity often emerges from intuitive, unconscious, and thus uncomputable, processes rather than rational and logical reasoning.

From a neurobiological point of view, novelty is the difference between what was previously predicted about a given object or situation and what actually happens (Shymkiv et al., 2025). To make such a conclusion, researchers conducted a study on the perception of sound in a mouse population, focusing on neuronal responses to expected and unexpected auditory stimuli. Imaging the auditory cortex has shown that neurons responded not just to sound but also to its novelty, leaving an “echo” that tracked sensory inputs over time. Thus, novelty can be seen here as a standard or automatic function of mammal brains. This aligns with the Bayesian brain hypothesis, which suggests that the brain continuously generates expectations about sensory input and updates them in response to discrepancies (Friston, 2010, p. 129). Technically speaking, some object or observation can be defined as novel “if it is a statistical outlier, meaning that it is significantly different from other members of the sample from which it is drawn” (Barto et al., 2013, p. 7).

However, divergent thinking does not directly indicate or measure creative thinking skills (Runco and Acar, 2012) but rather serves as a strong predictor among other factors (Hocevar, 1981): attitude & interest (motivation); personality inventories (traits); biographical inventories (life and creative experiences). In contrast to divergent thinking, convergent thinking focuses on finding a single, precise solution, such as solving a mathematical problem. This duality shows that creativity can emerge through both chaotic & self-organized (Schuldberg, 1999) and focused & strict ways of thinking. Both of them can be observed in neurobiological studies. While Rhodes’s 4P framework is fundamental for understanding creativity, recent advances in neuroscience suggest that this model may require refinement to capture the dynamic, network-based nature of creative cognition fully.

Using fMRI scans, Beaty et al. (2016) showed that divergent thinking engages the Default Mode Network (DMN), which is responsible for imaginative and spontaneous thought (Beaty, 2015). Moreover, active connectivity between the DMN and executive control networks (ECN) allows individuals to explore novel ideas while still applying goal-directed focus, which is mostly domain-specific (Jung et al., 2013). Exactly this interplay, between self-generated cognition (DMN) and evaluation of potential ideas in the within-goal-focus (ECN), is responsible for creative thinking, and thus the activity of these regions can predict how creative a thought or idea is (Beaty et al., 2015, 2018, 2019). Contrary to the more isolated dimensions in the 4P model, this view presents creativity as a fluid, multilayered system. According to researchers, the activation of the SN, which plays a role in switching between the default and control networks, is involved in this interplay. Early coupling between the DM and SN was interpreted as an intermediate switching mechanism that later facilitated the coupling between the default and control networks (Beaty et al., 2016, p. 3). This flexible switching, activated by the SN, is vital for the general understanding of the creative flow in cognition (Patti et al., 2024). More specifically, it enables fluidly alternating between spontaneous ideas and critical evaluation, by breaking and re-evaluating previous ideas and patterns and thus identifying which thoughts deserve attention and further cognitive investment (Picchi, 2025).

Consider Bayes’ theorem, which is used today from spam filters to sophisticated artificial intelligence algorithms in medicine, finance, and generally in statistical methods. It was developed by Thomas Bayes in the 17th century and remained unrecognized until Pierre-Simon Laplace re-evaluated and generalized his ideas - almost two centuries after Bayes’ death. Novelty is a pillar of any creative product, but its effectiveness may depend on time and scientific, technical, and social trends at the time of creation. The inventions and discoveries of Tesla and Mendel, Bruno and Galileo, the masterpieces of Vincent Van Gogh and Paul Gauguin - all of them (and many other creators) found their effectiveness only long after their creators had died. It was at the moment when novelty and efficiency converged that their ideas became the product of an incredible creative process.

## Definition of creativity

**Figure 2:**
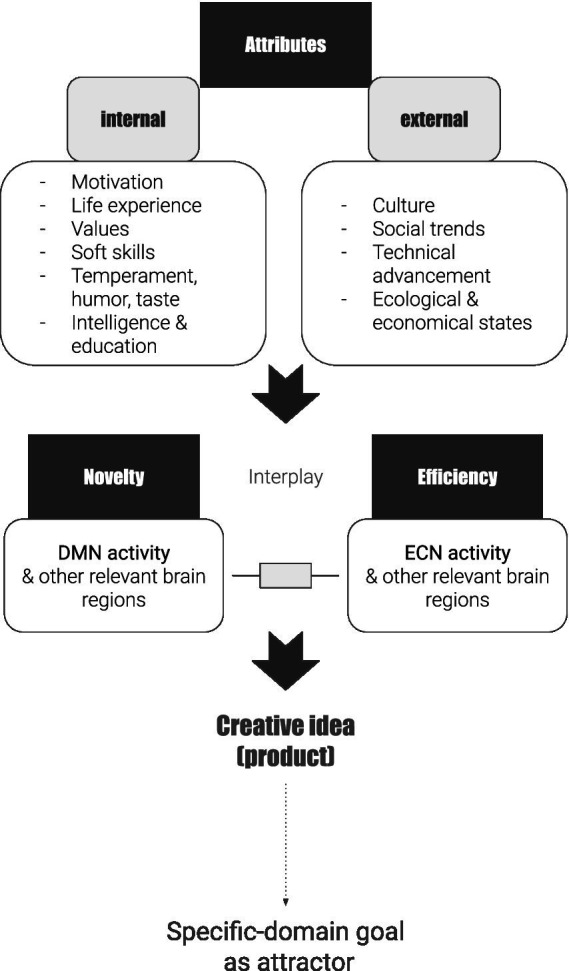
Extended model of creativity. Own conceptual model. DMN, Default Mode Network; ECN, Executive Control Network.

Based on the definitions and findings discussed above, the definition of creativity can be seen as an attempt to combine cognitive and neurobiological insights together and thus complementing each other. In this manner, creativity can be defined as a state of balance between chaotic (synonym: irrational, non-linear, uncontrolled, unpredictable, spontaneous, self-organized) and focused (synonym: rigorous, logical, centered, organized, filtered, disciplined), which is influences by internal and external attributes and attracted by a within-domain goal. Internal and External attributes in Figure 2 are presented as dominant examples, deriving from the studies above, and thus they are open to being extended by coming psychological, economic, and sociological studies. The DMN, ECN, and their interplay-salience network are derived from recent neurobiological studies. Attraction by a domain-specific goal is mostly inspired by Chaos Theory and its explanation of the brain as a nonlinear system with an existing attractor (Schuldberg, 1999; Díaz et al., 2015; Freeman, 1995; Skarda and Freeman, 1990; Tolchinsky, 2023) and complemented by studies of Baer (1998, 2012, 2015, 2016).

## Discussion: contribution of creativity to AI

“The technology is always an element of creativity. But it never is the source of the creativity.”

— Francis Ford Coppola

How are the concepts mentioned above relevant to AI? The connectivity between rational and irrational behavior can be seen in relativity. When a problem requires a probabilistic approach, the case of the conjunction fallacy indeed represents an irrational outcome. However, when a problem demands contextual sensitivity or real-life conditions, applying a purely probabilistic approach becomes irrational. Following this logic, creativity or creative solutions can be seen either as “irrational,” relating to the classical logic of solving a problem with a given set of components, or as rational, if the reference point is novelty within these components.

According to Boden (1998), there are three types of creativity that can be integrated into AI: (1) “by producing novel combinations of familiar ideas; (2) by exploring the potential of conceptual spaces; (3) by making transformations that enable the generation of previously impossible ideas” (p. 347). Even though most AI algorithms are based on probabilistic and divergent-thinking approaches, there have been a few breakthroughs in AI development toward some degree of creativity (Haase and Hanel, 2023; Guzik et al., 2023; Ramesh et al., 2022), not without some persisting limitations (Koivisto and Grassini, 2023; Grassini and Koivisto, 2024). Generally, these are the Transformer models (Vaswani et al., 2017) and the model of the Skill-Mix evaluation (Yu et al., 2023). Transformer models, which are used in ChatGPT, became successful due to their context-sensitivity and consequently more original output. Technically, transformers’ “creativity” can be tuned by the parameter of temperature, which is responsible for the “diversity” of the next predicted word in the prompt (Ficler and Goldberg, 2017; Holtzman et al., 2019). More specifically, temperature tuning controls the degree of randomness in generated outputs, surfacing between originality and coherence. This can be seen as a computational analog to the “Process” and “Product” dimensions in the 4P model: exploration (high temperature) mirrors divergent thinking, and exploitation (low temperature) aligns with convergent focus. However, a high diversity or unexpected result is sometimes unsuccessful compared to its quality (Hashimoto et al., 2019), i.e., it may be novel but less effective in a given context.

The skill-mix algorithm went deeper into understanding semantics itself and thus was able to catch the nuances of the semantic structure and apply the combination of its inner parameters—language skills, such as using metaphor, specific linguistic vocabulary, self-serving bias, etc. (Yu et al., 2023). In the context of the 4P model, this algorithm could be linked to the “Press” dimension, reflecting the role of environment and context. The researchers found that output generated using skill-mix evaluation gives an unexpected and efficient outcome that goes beyond predictions based on a training set. Indeed, these mechanisms simulate a fluid, context-sensitive integration of cognitive skills, similar to the dynamic interplay between DMN, ECN, and SN in the brain.

Following the extended model of creativity in Figure 2, this could indicate that establishing a more precise goal within a domain with its nuances could potentially lead to more meaningful and creative outcomes. Another hypothesis could be developed by operationalizing the internal and external attributes into such skills and testing how their manipulation/tuning affects the level of creativity of the result being generated. Such testing could provide a sharper view of what parameters might influence (or have no effect on) the final creative outcome, a problem described above by Laverghetta et al. (2025). Thus, applying this model opens at least two potential windows for exploring creativity in AI systems: the structure of domain-specific goals and corresponding parameters, and internal and external attributes as skills.

These examples demonstrate that understanding the mechanism of human creativity is essential for innovative AI performance, as it is created and evaluated by humans. Human creativity involves a dynamic interplay between divergent and convergent thinking, rational and irrational processes, and predictable and chaotic elements. It also spans dimensions such as fluency, flexibility, originality, and elaboration. While AI systems excel at generating numerous novel combinations and handling huge amounts of corresponding data, they cannot match humans’ embodied knowledge, emotional understanding, ethical reasoning, and intuitive leaps. The proposed model thus explores how these distinctly human creative qualities can guide AI design, aiming to develop systems that go beyond mere simulation to embrace a deeper understanding of creativity’s human foundations.
